# Population Genomics of Microbial Biostalactites: Non-recombinogenic Genome Islands and Microdiversification by Transposons

**DOI:** 10.3389/fmicb.2022.828531

**Published:** 2022-02-21

**Authors:** Kateřina Burkartová, Jiří Dresler, Jakub Rídl, Lukáš Falteisek

**Affiliations:** ^1^Department of Philosophy and History of Science, Faculty of Science, Charles University, Prague, Czechia; ^2^Military Medical Agency, Military Health Institute, Prague, Czechia; ^3^Department of Zoology, Faculty of Science, Charles University, Prague, Czechia; ^4^Laboratory of Genomics and Bioinformatics, Institute of Molecular Genetics, The Czech Academy of Sciences, Prague, Czechia; ^5^Department of Ecology, Faculty of Science, Charles University, Prague, Czechia

**Keywords:** chemolithotrophic bacteria, metagenome-assembled genomes, population genomics, mobile elements, horizontal gene flow

## Abstract

Intrapopulation genetic variability in prokaryotes is receiving increasing attention thanks to improving sequencing methods; however, the ability to distinguish intrapopulation variability from species clusters or initial stages of gene flow barrier development remains insufficient. To overcome this limitation, we took advantage of the lifestyle of *Ferrovum myxofaciens*, a species that may represent 99% of prokaryotic microbiome of biostalactites growing at acid mine drainage springs. We gained four complete and one draft metagenome-assembled *F. myxofaciens* genomes using Oxford Nanopore and Illumina sequencing and mapped the reads from each sample on the reference genomes to assess the intrapopulation variability. We observed two phenomena associated with intrapopulation variability: hypervariable regions affected by mobilome expansion called “scrapyards,” and variability in gene disruptions caused by transposons within each population. Both phenomena were previously described in prokaryotes. However, we present here for the first time scrapyard regression and the development of a new one. Nearly complete loss of intrapopulation short sequence variability in the old scrapyard and high variability in the new one suggest that localized gene flow suppression is necessary for scrapyard formation. Concerning the variable gene disruptions, up to 9 out of 41 occurrences per sample were located in highly conserved diguanylate cyclases/phosphodiesterases. We propose that microdiversification of life strategies may be an adaptive outcome of random diguanylate cyclase elimination. The mine biostalactites thus proved as a unique model system for describing genomic intrapopulation processes, as they offer easily sampleable units enriched in a single microbial species.

## Introduction

Population genetics was not initially applied to prokaryotic microorganisms since they were assumed as a simple cluster of clonally propagating lineages. Accumulation of diversifying mutations and occasional genome-wide selective sweeps are the only genetic processes allowed in this model. This idea was rejected after the discovery of widespread horizontal gene transfer (HGT) in prokaryotes. The genetic promiscuity among distantly related microbes created a picture of prokaryotic evolution as a network rather than tree. In this model, genomes could be imagined as transitory associations of phylogenetically diverse genes (e.g., Dagan et al., 2008). Increasing amount of closely related genomes available for a comparison led to revelation of genetically coherent microbial populations, where gene flow between lineages prevents their uncontrolled diversification by random mutations. In these populations, alleles combine freely and selection acts on individual genes instead of genomes in prokaryotes (Whitaker et al., 2005). Various models of functional genetic variability within natural microbial populations have been proposed. In a quasisexual model, alleles of many genes combine freely in a population and contribute to physiological fine tuning of individual cells within a wide ecological niche (Rosen et al., 2015). In a different model, physically connected, but genetically rather isolated subpopulations inhabit diverse microniches (Kashtan et al., 2014). Long-term observation of phylogenetically varied microorganisms from lake water revealed various degree of genetic coherence in different species. Here, the rates of HGT were usually sufficient to enable fixation of new alleles without genome-wide sweeps of variability. The genes thus dispersed by HGT independently to each other, similarly as in sexually reproducing organisms. Only a single genome-wide selective sweep was observed (Bendall et al., 2016).

Analyses of variant dispersal networks showed that intense HGT is restricted to specific groups of genotypes, while other microorganisms are excluded (Arevalo et al., 2019). These groups may overlap with makeshift “species” recognized by polyphasic taxonomy, but frequently are narrower. The gap between gene flow rates within and between groups was used to define prokaryotic biological species in a similar manner as for sexually reproducing organisms (Bobay and Ochman, 2017; Iranzo et al., 2019). Each HGT event sweeps single nucleotide polymorphisms (SNPs) that differentiated the two genomes in the affected stretch. In a group of closely related genomes that did not experience HGT, the SNPs are distributed randomly. A presence of unexpectedly long stretch without SNP indicates a recent HGT event in these genomes. Recombinogenic populations thus can be detected by a presence of identical genome regions that are longer than could be expected in case of random distribution of SNP. Recent studies showed that reduced gene flow between prokaryotic populations corresponds with ecological differentiation of these populations (Arevalo et al., 2019). It supports the paradigm that the coherent, or recombinogenic, groups have biological significance and are defined by an intrinsic self/non-self recognition rather than by a sequence similarity, gene content, or physical separation of the populations.

Our insight into the genetic structure of natural microbial populations suffers from serious methodological limitations. Culture-derived genomes can be obtained from a small proportion of culturable microorganisms only. The number of individual genomes is limited by the necessity to handle the cultures. These constrains can be partially overcome by single amplified genomes (SAGs); however, these genomes are usually incomplete and population coverage by SAGs is limited for technical reasons as well (Bendall et al., 2016; Garcia et al., 2018; Lopez-Perez et al., 2020). Sampling depth sufficient for quantitative assumptions can be reached by targeted sequencing of PCR amplified genetic markers, but this approach is restricted to previously selected genes or loci (Rosen et al., 2015). These methods are sufficient for gene flow network analyses and biological species delimitation, but their view of the total genetic variability is unavoidably simplified.

A promising approach is the mapping of shotgun metagenome reads on metagenome-assembled genomes (MAGs). High-quality MAGs are becoming common, but they usually represent an “average” genome of a microorganism, with no information comprising individual variability (Chen et al., 2020). The variability is then manifested in reads not fully homologous to the MAG. Depth of variability sampling is equal or higher than the coverage of the MAG. This approach is suitable for intrapopulation variability assessment, mainly in microbial communities with single or a few highly abundant microorganisms, which can be clearly differentiated from each other. In samples with high diversity, low sampling depth and greater error rates of the MAGs may be limiting (Meziti et al., 2021).

Free living single species-dominated microbial communities are rare. They can be found in deep underground environments limited by energy sources (Chivian et al., 2008), cyanobacterial blooms (Cai et al., 2014), and microbial streamers in acid mine drainage (Hallberg et al., 2006). In the present study, we investigated genetic variability of microbial communities that produce gelatinous biostalactites (i.e., snottites) at acidic water seepages in abandoned mines. These communities were dominated by the chemolithotrophic primary producer *Ferrovum myxofaciens*. It is the far most abundant stalactite-producing bacterium in dripwater with a pH range of 2.5–3 (Ziegler et al., 2009; Falteisek et al., 2016). Complete *F. myxofaciens* MAGs from biostalactites separated by distances from 10 to 500 km as well as from a single biostalactite resampled with a 2 years interval were compared. An overall genome conservation among these populations as well as intrapopulation variability patterns showed high importance of horizontal gene flow in *F. myxofaciens* genome maintenance.

## Materials and Methods

### Study Sites

The Mikulov mine is located in North Bohemia, Czech Republic. It was exploited for silver rich sulfide ores from the 15th century until 1858. The vein-type ore mineralization is composed of arsenopyrite and less common pyrite, sphalerite, galena, and younger carbonate minerals with native arsenic (Sattran, 1958). Quartz represents the main side mineral. The *Ferrovum*-rich biostalactites are confined to a single site where acid mine drainage (AMD) percolates from an old caved gallery. The samples MI1 and MI1A were physically connected by a gelatinous growth in a wooden gutter draining the caved gallery.

The exploratory adit (i.e., mine gallery connected to the surface) in Šobov quarry in Banská Štiavnica (central Slovakia) is a shallow adit from the 20th century, located in a massive metasomatic quartzite body around the outcrop of a huge polymetallic vein. Pyrite, galena, and sphalerite are dispersed in the host rock. Biostalactites are common at several sites with percolating acidic water within the adit.

Adit no. 112 in Oloví (North-west Bohemia) was built in 1950’s to explore a vein-type Pb-Ag deposit. Pyrite, arsenopyrite, sphalerite, and galena accompanied by quartz represent the main vein fillings. Abundant biostalactites grow in an area where the modern adit communicates with extensive late medieval – early modern times mine works.

For more detailed physicochemical characterization of the dripwater see Supplementary Table 1.

### Field Assessments

Stalactite dripping rates were calculated from filling times of a calibrated vessel. Physicochemical parameters of the dripwater (pH, ORP, EC, DOX, temperature) were determined using a portable multimeter Multi340i (WTW, Weilheim, Germany). Concentrations of Fe^2+^ and total Fe were determined by portable spectrophotometer DR3900 (Hach Lange, Düsseldorf, Germany) directly after sample collection. As (and other major cations) concentrations were determined by ICP-OES from filtered (<0.2 μm nylon membrane filter) and acidified (ultrapure HNO_3_) samples.

### DNA Extraction and Sequencing

Total genomic DNA was isolated using DNeasy PowerLyzer PowerSoil kit (Qiagen, Hilden, Germany) and checked by electrophoresis in 1% agarose gel in TBE. A typical smear of DNA sized > 10 kb was observed in all samples. Amplicons of SSU rDNA V4 region were sequenced on Illumina MiSeq platform at a depth of at least 10k sequences per sample to assess microbial diversity and relative abundance of *F. myxofaciens* in the biostalactites.

The Illumina sequencing libraries were prepared using TruSeq Nano DNA high-throughput kit. The protocol included the PCR amplification step. The fragmentation of DNA was performed on Bioruptor Plus sonication device. The quality of final libraries was checked on 2100 Bioanalyzer. The libraries were sequenced using the Illumina HiSeq RR 2 × 250 bp technology.

The WGA (Whole Genome Amplification) method was used to amplify the DNA for nanopore sequencing. Five μl of the total DNA was amplified using REPLI-g Mini Kit (Qiagen, Hilden, Germany) according to manufacturer’s instructions with the exception of 4 h incubation time. The amplified DNA was treated with T7 Endonuclease I (NEB) to resolve hyper-branched structures caused by the WGA. The barcoded nanopore sequencing libraries were prepared using Rapid Barcoding Kit SQK-RBK004 and sequenced with MinION instrument using FLO-MIN106 flowcell (Oxford Nanopore, Oxford, United Kingdom).

### Sequence Assembly

We trimmed the sequencing adapters from the nanopore reads using Porechop v0.2.4 software with ‘–discard_middle’ option^1^ and trimmed and quality filtered the Illumina data using Trimmomatic v0.39 with options ‘ILLUMINACLIP: < adaptor_fasta_file > :1:25:10, SLIDINGWINDOW:4:17, TRAILING:10 and MINLEN:100’ (Bolger et al., 2014). For each sample, the long nanopore reads were assembled using Flye v2.7.1 software with ‘-g 12m’ option (Kolmogorov et al., 2019). To further correct potential errors caused by a higher error rate of the nanopore technology we performed three rounds of a polishing step using BWA v0.7.17 package with command ‘bwa mem -M’ (Li, 2013) to map the Illumina reads on the corresponding draft assembly followed by a sequence correction in Pilon v1.23 software with ‘–fix all’ option (Walker et al., 2014). The genomes were annotated using RAST server (Aziz et al., 2008).

### Variant Calling and Data Analyses

Intrapopulation variability was analyzed individually for each sample. We took an advantage of the long nanopore reads to identify variants caused by transposable elements inside each sample. Minimap2 with options ‘–MD and -ax map-ont’ (Li, 2018) was used to map the nanopore reads on the corresponding genome assembly and the resulting mapping file was analyzed by variant caller software sniffles v1.0.11 with the following parameters: ‘-s 2, -l 10 and -r 1000’ (Sedlazeck et al., 2018) to identify larger structural variants in the nanopore data. The translated nucleotide sequences of the detected variants were aligned to NCBI-nr protein database using blastx v2.10.0 with ‘-evalue 0.1’ (Camacho et al., 2009). Variants with top hits to transposable elements entries were considered as transposon insertions.

Overall variability inside each sample was inferred based on the analysis of short nucleotide variants (including SNPs and other short variants spanning 2 or more bases). Here we mapped the Illumina data on the reference genome sequences using BWA v0.7.17 (Li, 2013) and performed the following variant detection using HaplotypeCaller command from the GATK v4.1.7 package (Poplin et al., 2017). Both commands were run under default settings.

Statistical significance of differences between scrapyards and the bulk genomes was tested in R software (R Core Team, 2019) using Mann–Whitney *U* test (Mann and Whitney, 1947).

### Phylogenetic Analyses

Sequences of 12 housekeeping genes (Supplementary Table 3) were picked manually from the published almost complete *Ferrovum* genomes (Ullrich et al., 2016) and from representative *Ferrovum* sp. MAGs deposited in NCBI Genome database. The sequences were translated and their concatenate was aligned using MAFFT (Katoh et al., 2019). Unaligned sites were trimmed manually and the phylogenetic tree was constructed in PhyMl v. 20120412 (Guindon et al., 2010) under LG gamma model with 1000 bootstrap replicates.

### Data Availability

The assembled genomes as well as raw sequencing data including nanopore and illumina reads have been submitted to the NCBI BioProject database^2^ under accession numbers PRJNA704679, PRJNA701483, PRJNA704691, PRJNA633238, PRJNA633240.

## Results

### The *Ferrovum myxofaciens* Genome From Metagenome

**FIGURE 1:**
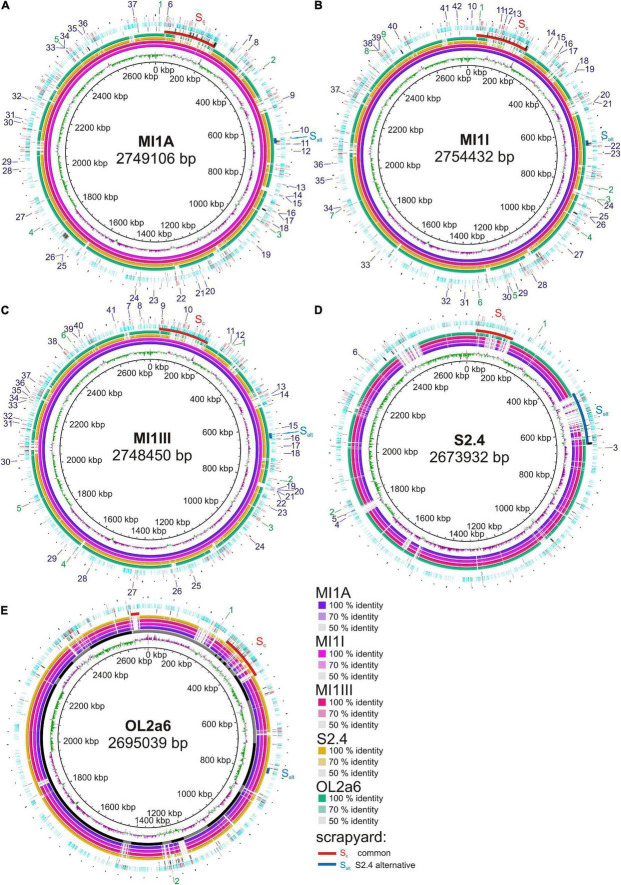
Reference-based whole genome comparisons of the new *Ferrovum myxofaciens* strains. One of the genomes was used as a reference in each ring: **(A)** MI1A, **(B)** MI1I, **(C)** MI1III, **(D)** S2.4, and **(E)** OL2a6. The innermost ring shows the GC skew. Four inner colored rings show the sequence identity between genomes from individual samples. Contig layout is displayed as black and gray sections in OL2a6 genome. Red and blue arcs mark the original and the S2.4 alternative scrapyard in each genome, respectively. The scrapyard is interrupted and GC skew is inconsistent due to random contig orientation in OL2a6. Three outer rings show (from the inside) (1) SSV variability revealed by Illumina read alternatives, (2) “scrapyard-like content” – marked are spots where the ORFs for hypothetical proteins (light blue), transposases or other MEs (red), and phage proteins (black) start, (3) presence/absence variability in insertion sequences (IS) revealed by Nanopore read alternatives: IS outside ORFs or inside hypothetical proteins (red). IS disrupting genes with annotated functions – IS is present in reference genome (green), IS is present in alternative reads (blues). The numbers of labels are described in Supplementary Tables 4a–e. The SSV variability is not available for genome MI1III, because Illumina reads are not available for this sample. The visualization was performed in BRIG (Alikhan et al., 2011) using Blastn method.

Based on 16S rDNA amplicon sequencing, 5 mine biostalactites with *Ferrovum* sp. representing 87.4–99.3% of the microbial communities were selected for metagenome sequencing (Supplementary Table 2). All biostalactites grew at AMD seepages located in abandoned mines. The basic characteristics as well as sample site locations are summarized in Supplementary Table 1. The assembly of Oxford nanopore sequence reads yielded circular scaffolds of *Ferrovum* genome in samples MI1A, MI1III, and S2.4. Two contigs, which have been joined manually using overlapping Illumina reads and formed circular scaffold, were obtained from sample MI1I. The *Ferrovum* sp. genome from sample OL2a6 was split into 25 contigs due to a low read coverage (Table 1). The contigs were then concatenated in a random order to obtain a draft genome. Basic characteristics of the genomes are summarized in Table 1. The GC skew suggests that all four complete genomes were assembled correctly (Figure 1).

**TABLE 1:** Basic characteristics of the five *Ferrovum myxofaciens* metagenome-derived genomes.

	Ferrovum SSU rDNA in amplicons (%)	Illumina coverage	Nanopore coverage	Contigs	GC content (%)	Length (bp)	Genes	tRNA genes	rRNA arrays
MI1A	87.4	1191	201.7	1	54.1	2749106	2751	42	2
MI1I	99.2	1320	169.5	1	54.1	2754432	2782	42	2
MI1III	88.4	0	401.0	1	54.1	2748450	2759	42	2
S2.4	98.9	1147	384.0	1	54.6	2673932	2670	42	2
OL2a6	98.4	1188	21.6	25	54.4	2695039	2722	42	2

### Phylogeny

**FIGURE 2:**
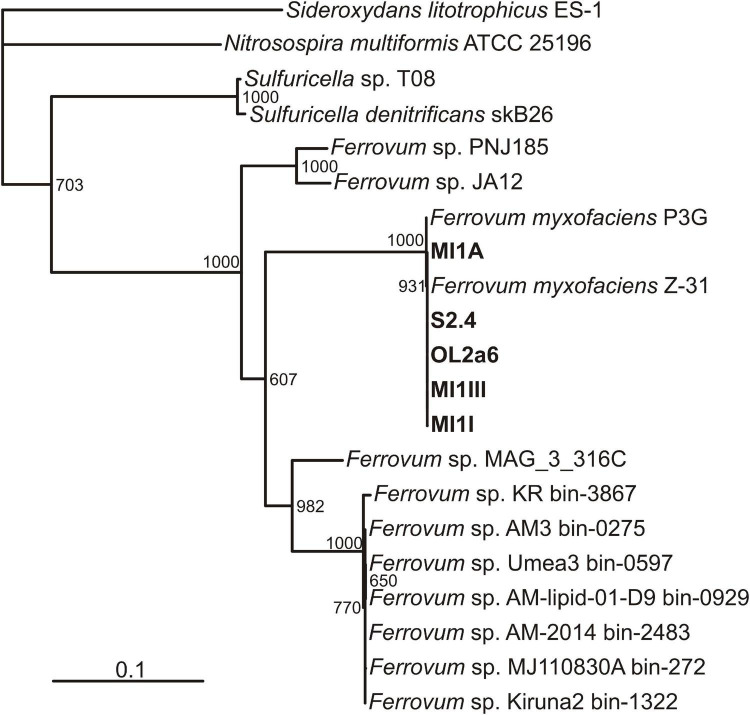
A phylogenetic tree based on 12 conserved housekeeping genes. New strains of *Ferrovum myxofaciens* described in this study are in bold.

All the circular genomes assembled have two copies of the small subunit ribosomal RNA gene (SSU rDNA) which shares a 99% similarity with the type strain *Ferrovum myxofaciens* P3G. Phylogenetic analysis of 12 conserved housekeeping genes (Supplementary Table 3) revealed a close relation of all samples to *Ferrovum myxofaciens* strain Z-31 and the type strain P3G (Moya-Beltrán et al., 2014; Ullrich et al., 2016; Figure 2). The genomes thus can be ascribed *to Ferrovum myxofaciens* as novel strains MI1I, MI1A, MI1III, S2.4, and OL2a6.

### Gene Content Variability

The gene content was highly conserved among the three samples from Mikulov. Only two genes with unknown functions were unique for a single sample. Due to negligible gene content variation we will henceforth use the name “MI group” for the genomes MI1A, MI1I, and MI1III.

It was possible to distinguish two types of gene content differences between genomes from various sites (Figure 1). The first type is represented by groups of genes or operons that encode proteins with some accessory biological function. The second type includes large arrays enriched in genes with unknown biological function (hypothetical proteins, HP) and genes not encoding adaptive biological functions that can be characterized as selfish DNA [e.g., phage proteins, mobile elements (ME), restriction-modification systems, toxin-antitoxin systems].

The most notable metabolic variation between strains from different sites was in the presence of the carboxysome operon. All *F. myxofaciens* strains had a complete CO_2_ pathway using the RUBISCO operon. Strains S2.4 and OL2a6 possessed two paralogs of the RUBISCO operon – one with a complete carboxysome gene cluster and one without it. MI group members lacked the entire carboxysome gene cluster. Further differences in the presence of encoded metabolic functions comprised assimilatory nitrate reduction, cellulose degradation/synthesis and mercury resistance. These accessory metabolic functions had a mosaic distribution across the strains as no strain possessed all of them (Table 2).

**TABLE 2:** The main differences in content of genes with known function in *Ferrovum myxofaciens* from various sites.

Operon/gene cluster	MI group	S2.4	OL2a6	Position in genomes (kbp)	Length (kbp)
Cellulose biosyntheses/degradation	Yes	No	No	823–835 (in MI1A)	12
Assimilatory nitrate reduction	Yes	Yes	No	1287–1302 (in MI1A)	15
Carboxysome gene cluster	No	Yes	Yes	1586–1591 (in S2.4)	5
Mercury resistance and reduction	No	Yes	No	467–470 (in S2.4)	3
CRISPR-Cas system type I	No	Yes	No	1525–1534 (in S2.4)	9

Important non-metabolic differences concern the CRISPR-Cas system. Each strain possessed a type III CRISPR-Cas system, but a functional CRISPR-Cas system type I could be found in the S2.4 strain only. A residuum of the CRISPR-Cas system I – a disrupted exonuclease Cas4 – was present in the genomes OL2a6 and the MI group.

### Genome “Scrapyards”

The second type of variability included genes encoding HPs and selfish DNA elements. Approximately 40–45% of the HPs were assigned to protein families using combination of annotations, while two thirds of them were mobilome-related proteins. This content accumulated in some regions, while in other regions it was sparse. Due to the presumably “unfavorable” content of these regions we call them scrapyards. The main scrapyard had been found at identical position in MI group and OL2a6 genomes (Table 3 and Figure 1 – red arcs). In OL2a6, the putative scrapyard was split into 9 contigs covering the range 293 – 450 kbp of the concatenate (Figure 1E). Several shorter scrapyard-like arrays were present throughout the genomes.

**TABLE 3:** Basic characteristics of “scrapyard” genome region of five *Ferrovum myxofaciens* strains.

	Scrapyard																	
	Hypothetical proteins			Phage protein			ME proteins			Rest of ORF			SSV					
						
	Count	%	Per 10 kbp	Count	%	Per 10 kbp	Count	%	Per 10 kbp	Count	%	Per 10 kbp	Count	Per 10 kbp	ORF total	Length (bp)	From bp	To bp
MI1A	175	71.7	9.03	9	3.69	0.46	22	9.02	1.13	38	15.6	1.96	231	11.9	244	193839	32354	226193
MI1I	175	70.3	9.04	10	4.02	0.52	21	8.43	1.09	43	17.3	2.22	162	8.37	249	193534	32354	225888
MI1III	162	71.1	8.79	9	3.95	0.49	16	7.02	0.87	41	18.0	2.22	N/A	N/A	228	184321	32348	216669
S2.4	95	61.7	7.05	1	0.65	0.07	7	4.55	0.52	51	33.1	3.78	3	0.22	154	134793	31234	166027
OL2a6	142	66.1	9.07	0	0	0	32	14.9	2.04	41	19.1	2.62	61	3.89	215	156636	293058	449694
S2.4 alt.	150	62.2	7.97	18	7.47	0.96	12	4.98	0.64	61	25.3	3.24	278	14.8	241	188208	456809	645017

	**Outside scrapyard**																	
	**Hypothetical proteins**			**Phage protein**			**ME proteins**			**Rest of ORF**			**SSV**					
	**Count**	**%**	**Per 10 kbp**	**Count**	**%**	**Per 10 kbp**	**Count**	**%**	**Per 10 kbp**	**Count**	**%**	**Per 10 kbp**	**Count**	**Per 10 kbp**	**ORF total**	**Length (bp)**		

MI1A	1026	37.4	4.02	13	0.47	0.05	116	4.22	0.45	1592	58.0	6.23	452	1.77	2747	2555267		
MI1I	1040	37.6	4.06	12	0.43	0.05	127	4.59	0.50	1588	57.4	6.20	413	1.61	2767	2560898		
MI1III	1027	37.4	4.01	12	0.44	0.05	118	4.30	0.46	1590	57.9	6.20	N/A	N/A	2747	2564129		
S2.4	853	34.2	3.63	12	0.48	0.05	49	1.96	0.21	1522	61.0	6.47	317	1.35	2497	2350931		
OL2a6	1030	37.0	4.06	8	0.29	0.03	169	6.07	0.67	1576	56.6	6.21	355	1.40	2783	2538403		

*The “scrapyards” are delimited by conserved positions of tRNA-Ser-CGA and tRNA-Val-CAC genes in each genome.*

The number of HPs, phage proteins and ME inside and outside scrapyards is shown in Table 3. Outside the scrapyard the concentration of HPs was 1.7–1.9 times lower (*p* = 0.012) and the concentration of ME-related proteins was 1.6–2.5 times lower (*p* = 0.016). On the contrary, the concentration of genes with adaptive biological function was 1.8–3.7 times higher outside the scrapyard (*p* = 0.008). Phage proteins were more concentrated in scrapyards of the MI group only. GC skew was strikingly scattered in all scrapyard regions (Figure 1).

For the intrapopulation short sequence variability (SSV) assessment we mapped Illumina reads on the assembled reference genomes. The SSV was represented mostly by 1 bp substitutions and indels. Sporadic longer SSVs up to 139 bp were found. The SSV data are missing for MI1III due to absence of Illumina reads. A striking phenomenon was the concentration of SSVs in the scrapyards (Figure 1 and Table 3). The concentrations of SSVs were 6.7x, 5.2x, and 2.8x higher in scrapyards than in the rest of MI1A, MI1I, and OL2a6 genomes. However, in S2.4, the SSV concentration was 6.1x lower in this region compared to the rest of the genome. Another region spanning from 457 to 645 kbp fitted more accurately the scrapyard definition in S2.4. This region length expanded from ca 20 kbp (MI group) to 188 kbp in S2.4 and contained a 136 kbp island rich in selfish DNA with no synteny to the other strains (Figure 1D). Mercury resistance is the only adaptive feature encoded in the unique region (Table 2). The concentration of SSVs in the alternative scrapyard was 11.5x higher than in the rest of the genome (*p* = 0.057 with S2.4 alternative scrapyard). Concentration of HP, phage proteins, and ME exceeded the values of the original scrapyard in S2.4 genome (Table 3). GC skew was fluctuating in both scrapyards of S2.4. Two regions inside the alternative scrapyard of S2.4 mapped to different positions in the MI group genomes and were missing in OL2a6. The larger one was 18 kbp long, contained genes for assimilatory nitrate reduction, and was located at 1283–1302 kbp in MI1A. The second region was 12 kbp long and lied inside the original scrapyard in the MI group.

The proportion of SSV lying inside open reading frames (ORF) was 86, 81, 87, and 85% in MI1A, MI1I, S2.4, and OL2a6, respectively. Most SSVs were located in ME-related proteins and circadian clock protein KaiC in all genomes (Supplementary Table 6). A heavy metal efflux operon (CusA, CusB, and CzcB) was affected in S2.4. SSV-rich arrays usually spanned over more successive genes. Their borders were located inside as well as between ORFs.

Comparing the SSV layout in the MI group genomes, there was a region with SSV accumulation present in MI1A but absent in MI1I. In MI1A, this region lied between ca 1720 and 1752 kbp and contained 34 SSVs. The SSV here was distributed between 16 genes including 5-methyltetrahydrofolate-homocysteine methyltransferase, two genes related to nitrogen metabolism, three to the biosynthesis of pyrimidines, and seven HPs.

### Gene Disruption Variability

Transposons and other MEs were common in all *Ferrovum myxofaciens* genomes (Figure 1). 137, 147, 133, 67, and 200 genes were annotated as transposases or ME proteins in MI1A, MI1I, MI1III, S2.4, and OL2a6 genomes, respectively. The Oxford Nanopore technology provides reads long enough to span whole ME together with flanking sequences. We mapped the nanopore reads on the assembled genomes to identify sites, where both versions with and without ME insertion are present within a single population. Only variants in which at least two reads corresponded to the less common variant were considered as variable. Multiple variable MEs were identified in all genomes. Significant portion of them interrupted ORFs of various genes. The gene disruptions found by automated search are described in Supplementary Table 4 and visualized in Figure 1.

MI1A, MI1I, MI1III, S2.4, and OL2a6 genomes had 182, 202, 193, 56, and 5 variable MEs, while 37, 42, 41, 6, and 2 out of them disrupted protein coding genes, respectively. Disruptions of MEs and phage proteins as well as variable ME insertions to intergenic sequences were not included. Strains of the MI group had higher variability in disrupted genes than S2.4 and OL2a6. The disruptions were distributed rather evenly across the genome, apart from the region from 1400 to 1800 kbp with fewer gene disruptions in the MI group. This region also contained fewer phage proteins, MEs, and HPs than the rest of the genome. 9 of 37 variable gene disruptions were located in genes encoding GGDEF domain-containing diguanylate cyclases (DGC) in MI1A. In the other genomes, DGCs were also the genes most often attacked by MEs (Supplementary Table 4). In total, 18 highly sequence-conserved DGS paralogs were present in all genomes, 1 paralog was unique for the MI group and 2 for OL2a6. Gene duplication was observed in S2.4. Among the conserved DGCs, 10 paralogs were disrupted at least in one genome (Table 4). Genes related to motility, cellulose metabolism and assimilatory nitrate reduction were often disrupted in the MI group genomes. Selfish DNA elements were variably disrupted as well. Two independent disruptions of a single gene were detected four times (Supplementary Table 4): twice a DGC, RcpA, and DNA helicase RecQ.

**TABLE 4:** List of genes encoding diguanylate cyclases (contain GGDEF and EAL domains).

		MI group			S	OL
No.	l (bp)	1I	1A	1III		
1	1812	1v	1n	1n	0	0
a	2205	1v	1n	1n		
2	2046	0	0	0	0	0
3	2100	1v	1v	1v	1v1m	0
b	2619	0f	0f	0f	0	0f
4	3081	2v	1v	1m	0	0
c	1842				0	0f
5	2568	0	0	0	0	0
6	921	0	0	0	0	0
d	1896	0	0	0		0
7	2283	1v	1v	0	0	0
8	1701	1v	1v	1v	0	0
9	1194	0	0	0	0	0
10	1236	0	0	0	0	0
11	3003	0	0	0	0	0
12	2760	1v	2v	1v	0	0
13	2871	0	0	0	0	0
14	2100	0	0	0	0	0
e†	2797				0	
15	2649	0	1v	1v	0	0
16	2094	0	1v	1v	0	0
17	2145	1v	1v	1v	0	0
18	1692	0	0	1v	1m	1?‡
f	192	0	0	0		0
g	2403	0	0	0		
h	936					0
i	1038					0

*Genes conserved in all samples are marked by numbers 1-18, sequences missing or fragmentary in a part of samples are marked by letters a-i. Numbering of the genes correspond to their order in MI group genomes (position of genes c and e was inferred from surrounding sequences).*

*n, possibly non-variable disruption by ME.*

*v, variable disruption by ME.*

*m, disrupted by nonsense mutation.*

*f, fragment of a gene (partially lost).*

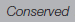
.

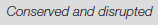
.

*†, Duplication of DGC3.*

*‡, Contig end.*

In order to eliminate the possibility that the variable gene disruptions were an assembly artifact, we performed PCR control for six selected genes (Supplementary Table 5). Representatives of each disrupted and intact gene were further confirmed by Sanger sequencing. Concurrent presence of a disrupted and intact gene in a single sample was proven by this assembly independent method in 13 cases. Two disruptions were detected by PCR only. The PCR did not detect the disruption found in Nanopore reads in five cases. Good agreement of PCR and Nanopore was obtained in 18 cases.

We computed the ratio of disrupted:intact gene variants for eight genes using the high-coverage Illumina reads. The sequences of the ME insertion boundaries were searched in Illumina reads by BLAST and the coverage of both variants was calculated as disruption ratio (Table 5). The variability of disruptions was confirmed in 15 cases in accordance with the Nanopore data. One variable disruption was not confirmed by the Illumina reads (Table 5). The intact versions of all except one gene predominated in the Illumina reads (disruption ratio < 1). Variable disruptions at two different sites were established in the RcpA gene by Illumina data only. Concurrent presence of both MEs in the gene was not observed despite the high number of reads covering the adjacent insertion sites.

**TABLE 5:** The Illumina reads quantity in eight genes afflicted by IS disruption.

	MI1A		MI1I		S2.4		OL2a6	
Gene annotation	nr.	Disruption ratio	nr.	Disruption ratio	nr.	Disruption ratio	nr.	Disruption ratio
Aerotaxis sensor receptor	**6**	y/n = 0.32	**1**	y/n = 0.45	**x**	no gene	**x**	n; contig end
Acetate kinase	**14**	y/n = 0.54	**2**	y/n = 0.64	**x**	n (0/114)	**x**	n (0/104)
Cellulose biosynthesis regulation protein BcsB	**15**	y/n = 0.28	**3**	y/n = 0.56	**x**	no gene	**x**	no gene
Diguanylate cyclase/phosphodiesterase DGC8	**22**	y/n = 0.74	**5**	y/n = 0.61	**x**	n (0/133)	**x**	n (0/117)
Type II/IV secretion system secretin RcpA/CpaC; disruption 1	**5**	y/n = 0.94	**8**	y/n = 1.48	**x**	n (0/45)	**x**	n (0/75)
Type II/IV secretion system secretin RcpA/CpaC; disruption 2	**5**	y/n = 0.4	**8**	y/n = 0.78	**x**	n (0/83)	**x**	n (0/131)
Uptake [NiFe] hydrogenase, small subunit HyaA	**x**	n (0/88)	**x**	n (0/102)	**x**	n (0/78)	**x**	n (0/73)
Diguanylate cyclase/phosphodiesterase DGC3	**x**	n (0/129)	**14**	n (0/150)	**1**	y/n = 0.78	**x**	n (0/124)
Diguanylate cyclase/phosphodiesterase DGC4	**2**	y/n = 0.52	**17, 18**	y/n = 0.44	**x**	n (0/86)	**x**	n (0/126)

*The columns marked “nr.” are marked identically as in Figure 2 and Supplementary Table 4. The column “disruption ratio” shows the ratio of disrupted/complete Illumina reads found manually using blastn. The symbol “n” indicates that no reads corresponded to disrupted gene (total numbers of analyzed reads are given in parentheses). If the gene is missing in the reference genome it is marked as “no gene.”*

## Discussion

### The Population Genomics of Simple Microbial Communities

We used a combination of long-read and short-read sequencing methods for *de novo* assembly of complete *Ferrovum myxofaciens* genomes from metagenomes. Subsequent backward mapping of the sequence data on these reference genomes was used to assess the intrapopulation genetic variability of the bacterium. Besides the advantage of the combination of sequencing methods, specific properties of the model organism enhanced the intrapopulation variability-targeted analysis. *F. myxofaciens* represents a dominant life form in confined low-diversity communities (Hallberg et al., 2006). This feature enabled us to perform genome assembly without any binning or cultivation steps. *Ferrovum* represents the only known genus of *Ferrovales*. While an increasing number of genotypes are being detected within this genus, they are genetically and ecologically distinct from *F. myxofaciens* (Ullrich et al., 2016; Grettenberger et al., 2020; Plewniak et al., 2020). A deep 16S rDNA amplicon sequencing revealed a single other *Ferrovum* sequence variant in the present five samples. This microorganism was extremely rare, representing 0.01–0.39% of the microbial communities. A preliminary genomic assembly revealed an average nucleotide identity of conserved genes (ribosomal proteins, transcription factors) with *F. myxofaciens* reaching 70–82% only (data not shown). False mapping of this species on the *F. myxofaciens* genome is thus improbable. Additional beneficial traits of the biostalactites result from their physical character (Ziegler et al., 2009; Falteisek et al., 2016). Each biostalactite represent a macroscopic continuous habitat without internal dispersal barriers, while biostalactites located at multiple spots of AMD dripping or in one or more mines can be clearly considered as distinct “islands,” hosting separated *F. myxofaciens* populations with various degrees of physical separation from each other.

The approach using long reads from simple communities is complementary to other strategies used in population genomics. In the studies based on mapping of metagenomic reads on multiple MAGs or SAGs, competitive recruitment of the reads to the genomes was required due to presence of closely related populations in the samples (Bendall et al., 2016; Garcia et al., 2018; Lopez-Perez et al., 2020). This approach is efficient for time-lapse observation of population heterogeneity or SAGs relative abundance. However, false variability caused by inclusion of reads derived from another population, which is not represented by SAG, as well as erroneous rejection of reads which map to highly variable regions (e.g., scrapyard) may occur. Sensitivity for long variants, i.e., the variable insertions of MEs should be significantly reduced in SAGs due to assembly artifacts or contig breaks occurring at sites containing repetitive sequences longer than a single Illumina read.

In the studies comparing multiple genomes from cultures or individual cells, rare variants can be easily omitted. Hypervariable regions with suppressed recombination can be easily overlooked or misinterpreted as a few distinct alleles due to low depth of sampling. On the other hand, association of specific variants throughout the individual genome can be resolved by the culture or single cell-based methods only (compare with, e.g., Cadillo-Quiroz et al., 2012).

### Intrapopulation Variability of *Ferrovum myxofaciens*

Two conspicuous patterns can be recognized in the *F. myxofaciens* population genetic structure. First, a highly unequal distribution of SSV associated with the expansion of suspect parasitic genetic elements in the genomic scrapyards, and second, variability in the prevalence of mobile elements and gene disruptions caused by their insertions.

All *F. myxofaciens* populations were markedly uniform, having hundreds of SSVs only. In the ecologically comparable facultative autotroph *Sulfolobus islandicus* from a single hot spring, 8185 single nucleotide substitutions were identified from 12 culture-derived genomes (Cadillo-Quiroz et al., 2012). The variability may differ significantly in various bacteria from the same environment and even in a single population at various time points probably due to ongoing selective sweeps (Bendall et al., 2016). However, the distribution of variability in *F. myxofaciens* genomes excludes a recent genome-wide sweep. SSVs are confined to specific regions, while several entirely SSV-free sections reaching 250–300 kbp were found. The SSV-rich regions start and end sharply and do not respect gene and operon boundaries. Presence of long homogeneous sections dispersed in a generally variable genome is a typical result of active horizontal gene flow (HGT). Length excess of SSV-free stretches is even used to search recombinogenic microbial genomes (Arevalo et al., 2019).

This poses a hypothesis that SSVs at the variable stretches of *F. myxofaciens* genome represent intrapopulation variability generated by random mutations that accumulated after last genome-wide selection sweep or bottleneck of the population. These mutations were swept by HGT on most of the genome and we can observe them at sites with suppressed HGT only. The patterns of SSV distribution are in good agreement with a quasisexual model of microbial populations (Rosen et al., 2015). Similar patterns were detected using SAGs in the cyanobacterium *Prochlorococcus* sp. SSVs making differences between distinct populations were dispersed among the entire genome, while intrapopulation variability was confined to variable islands (Kashtan et al., 2014). Region-specific HGT barriers mediated by a yet unknown mechanism have been proposed to explain the variability patterns of various prokaryotes including *S. islandicus* and *Vibrio cyclitrophicus* (Cadillo-Quiroz et al., 2012; Shapiro and Polz, 2014).

### The Genomic Scrapyards

A scrapyard differs from the rest of the genome by multiple parameters that signify for accumulation of neutral SSVs together with expansion of selfish DNA, fluctuating GC skew, and decrease of coding capacity. These phenomena resemble the well-known deterioration of heterogametic gonosomes in sexually reproducing organisms (e.g., Y chromosome in mammals). The scrapyard was located at the same position in all genomes of the MI group and OL2a6. In S2.4, the respective region has a reduced length and lacks almost all SSVs, while the other scrapyard features are partially preserved. Another genome island showing all scrapyard features was found in S2.4 genome. Several shorter regions with incomplete scrapyard characteristics can be found in all genomes.

We propose local suppression of HGT as the main causal factor in genesis of the scrapyard regions in *F. myxofaciens*. The mechanism of the suppression probably depends on high intrapopulation variability of scrapyards. However, it cannot be explained by a mere divergence of sequences, since the minimum length of identical DNA stretches for homologous recombination is 20–22 bp only (Grogan and Stengel, 2008). By this hypothesis, *F. myxofaciens* S2.4 represented a population similar to the MI group that passed through a bottleneck. During this bottleneck, possibly associated with dispersal of a few cells to a new locality, intrapopulation variability of the original scrapyard was lost and HGT in this area was renewed. Instant loss of entire SSV and gradual suppression of deleterious gene content is an expectable outcome of this process. A new scrapyard established randomly after the bottleneck event.

A genome island similar to *F. myxofaciens* scrapyards has been described in phylogenetically distinct bacteria including *Roseococcus*, *Salinibacter*, and *Myxococcus* (Wielgoss et al., 2016; González-Torres and Gabaldon, 2018; Wang et al., 2020). The authors propose several functions of this region, including gates for gene exchange with unrelated microbial lineages, initial genetic divergence of populations, self/non-self discrimination and accumulation of mobilome. In *F. myxofaciens*, SSV rather than variability in gene content was observed. Additionally, accumulation of MEs, prophages and RMS rather than potentially beneficial accessory genome takes place in the scrapyards. We thus prefer the neutral theory of scrapyard genesis by suppression of homologous recombination and subsequent deliberate expansion of selfish DNA, although a function of scrapyards as a sink for deleterious genetic material can not be ruled out. It can potentially be an intriguing model for investigation of mechanisms suppressing HGT in a region- or sequence-specific manner.

### Mobile Elements Generate Functional Diversification

The variability in gene disruption affects tens of genes in each population. A genome assembly artifact was excluded by several independent ways including targeted PCR amplification of selected variable disruptions. PCR revealed variable disruptions even in samples in which metagenomics did not. We explain this phenomenon by the high sensitivity of PCR that can detect sequences which were missed or underrepresented in the metagenomes. It should be noted that the variability revealed by population genomics is reliable, but this approach cannot be used to disprove the presence of a specific variant since rare variants may be randomly missed in the metagenome. It probably explains the low amount of ME-related variability in sample OL2a6 with low coverage.

Functional interpretation of the ME-related variability is not clear. Experiments with artificially activated ME showed that only ca 12% of genome may not be interrupted in laboratory culture of *Caulobacter crescentus* (Christen et al., 2011). Although life in culture requires a lower physiological plasticity compared to natural environment, and the genome of *C. crescentus* is more complex than that of *F. myxofaciens* and thus may be more redundant, we consider that the variable ME insertions can easily be not lethal for the affected *F. myxofaciens* cells. The repertoire of disrupted genes is non-random, with a striking prevalence of dispensable metabolic enzymes and regulatory proteins, mostly DGCs. Many of the affected metabolic pathways are missing in some *Ferrovum* sp. strains (Ullrich et al., 2016). However, all disrupted DGCs but one share perfect sequence homology among all *F. myxofaciens* populations. It suggests that we do not observe evolutionary perishing of these genes. A more complex explanation of ME dynamics is thus required.

The simplest explanation proposes random disruption of genes and survival of lineages where only dispensable genes were affected. A mechanism avoiding gene loss should be proposed, since otherwise the high number of concurrently affected genes would lead to unreasonably high rates of genome erosion. A community-based genome maintenance including regain of intact genes by homologous recombination and positive selection of less eroded genome variants seems to be the simplest mechanism. The presence of ca 200 variable MEs and 40–50 gene disruptions in a population may represent a steady state, in which the rate of new ME insertions is in equilibrium with reparation. Thus, the observed variable disruptions represent results of the most recent sublethal ME insertions which have not yet been eliminated. An occasional disruption of genes belonging to “selfish DNA” and their subsequent loss instead of reparation is compatible with the random model.

However, the random model cannot explain the selectivity of gene disruptions. Most notably, 19.5% (nine cases) of disrupted genes are DGCs in MI1I (and 24% in MI1A when two possibly non-variable disruptions are included). All the disruptions but one are located in highly conserved DGCs found in all strains. As an example, a low dN/dS ratio of 0.36, which indicates purifying selection, was found in the double disrupted DGC12. It indicates that variable disruptions are commonly located in genes, whose loss or deleterious mutations are not tolerated. Two different disruptions of the same gene within the same population were observed four times. When a possibly non-variable disruption of cardiolipin synthase in OL2a6 is included, three cases of unrelated disruptions of the same genes in populations from different sites have been detected. It represents one third of the disruptions detected in S2.4 and OL2a6. The homoplastic disruptions can be explained either by an extremely small pool of dispensable genes in *F. myxofaciens*, which is incompatible with the findings of Christen et al. (2011), or by a non-random pattern of gene disruption.

Insertion of a ME can result in an inactivation of the disrupted gene as well as activation of the surrounding genes (Vandecraen et al., 2017). It offers a possibility that a long-term gene activity modulation by the variable ME insertions diversifies the genetically uniform population. Loss of genes for synthesis of a beneficial compound, which does not have to be produced by all cells, in a part of a population is often associated with the “social cheating” life strategies (Cordero and Polz, 2014). Variable disruptions of assimilatory nitrate reductase and genes encoding cellulose degradation complex may belong to this type of strategy. Enzymes with DGC activity regulate bacterial behavior in many important processes, including switching between formation of biofilm and swarmer cells (Ferreira et al., 2008). It was demonstrated on various microbes that mutants with artificially disrupted DGCs had different salt concentration tolerance, showed abnormal heterocyst formation, or failed to form biofilms (Neunuebel and Golden, 2008; Dogra et al., 2013; Nascimento et al., 2014). Disruption of various DGCs can lead to a diversification of *F. myxofaciens* life strategies. These strategies may include competitive strategies (e.g., colonization of new sites vs. competition; Denef et al., 2012) as well as fine tuning to physicochemically diverse microhabitats. Ziegler et al. (2013) found a sharp oxygen gradient within the mine biostalactite. The Fe^2+^ concentrations differ by more than an order of magnitude between the analyzed samples, including the adjacent stalactites MI1I and MI1A. A possibility of physiological fine tuning to diverse conditions thus would be adaptive for *F. myxofaciens*. Additional support for the strategy diversification hypothesis is the fact that DGC disruptions are stable in time. Similar disruptions are in samples MI1I (plus MI1A) and MI1III. The minor differences can be explained by a limited sensitivity of the analysis (Table 4). Thus, natural selection during 2 years and three regenerations of the MI1 stalactite biomass (it was cut away at the base twice between MI1I and MI1III collection) did not eliminate the disruptions. On the contrary, the various disruption-bearing lineages propagated concurrently into the newly growing stalactite.

For these reasons, we believe that at least some of the ME-related variability is adaptive. Disrupted and intact forms of tens of important genes persist concurrently in *F. myxofaciens* communities. Presence of cells possessing all core genes in an intact form seems unlikely although we can not rule it out based on our data. A community of cells varying in the spectrum of disruptions is thus needed to maintain intact versions of all core genes. Alternatively, the disrupted genes can be rescued by spontaneous excision of ME. This hypothesis is not in conflict with occasional deleterious ME insertions nor with the silencing or elimination of unwanted genetic content by MEs.

## Conclusion

We established a population genomic approach which enables to detect and quantify genetic variability in natural microbial populations. The approach is based on the mapping of metagenomic reads on metagenome-assembled microbial genomes. Unlike previous studies, no arbitrary delimitation of populations was used due to the selection of simple and spatially distinct communities dominated by *Ferrovum myxofaciens*, which differ sufficiently from other community members. Long read-producing Oxford Nanopore sequencing was used to resolve ambiguities caused by repetitive sequences and to assemble complete genomes from metagenomes.

Low intrapopulation sequence variability (mostly SNPs) was found within the *F. myxofaciens* genome. Most variability was confined to genomic islands called “scrapyards,” characterized by low content of core genes, accumulation of suspect detrimental genetic elements, and inconsistent GC skew. A reduction of the original scrapyard and formation of a new one at a different location was observed in one *F. myxofaciens* strain. The observations are consistent with a quasisexual model of the population. Selective and reversible suppression of horizontal gene flow by an unknown mechanism took place in the scrapyard regions.

Disruption of multiple genes by mobile elements has been detected, while both disrupted and intact versions of up to 41 genes were detected within a single population. Conserved regulatory proteins, mostly DGCs, and dispensable metabolic enzymes are enriched among these genes. Instant diversification of life strategies and ecological fine tuning without the need of gene gain or loss may be the result of these disruptions. Since multiple variably disrupted genes belong to the core genome, most *Ferrovum* cells do not possess intact versions of all core genes. Thus, a community-based rather than individual maintenance of the genome is proposed.

Further research including a substantially greater number of samples should be performed. An advantage of *F. myxofaciens* biostalactites is that each stalactite can be treated as an island-like population with a variable degree of isolation from the others. The stalactites occur in great numbers in abandoned mines. Deeper insight in the patterns of selective gene flow suppression and functional interpretation of this widespread phenomenon can be the most significant benefit from the population genomics of simple natural microbial communities.

## Data Availability Statement

The datasets presented in this study can be found in online repositories. The names of the repository/repositories and accession number(s) can be found below: NCBI BioProject database (https://www.ncbi.nlm.nih.gov/bioproject/), PRJNA704679, PRJNA701483, PRJNA704691, PRJNA633238, PRJNA633240.

## Author Contributions

LF and KB designed the study, collected the samples, and wrote the manuscript. KB performed the Illumina sequencing. JD and JR performed the nanopore sequencing. JR assembled the genomes and conducted the bioinformatic analyses. LF performed the PCR tests. All authors contributed to the article and approved the submitted version.

## Conflict of Interest

The authors declare that the research was conducted in the absence of any commercial or financial relationships that could be construed as a potential conflict of interest.

## Publisher’s Note

All claims expressed in this article are solely those of the authors and do not necessarily represent those of their affiliated organizations, or those of the publisher, the editors and the reviewers. Any product that may be evaluated in this article, or claim that may be made by its manufacturer, is not guaranteed or endorsed by the publisher.

## Footnotes

1
https://github.com/rrwick/Porechop

2
https://www.ncbi.nlm.nih.gov/bioproject/
