# Supplementary material for: Population Genomics of Microbial Biostalactites: Non-recombinogenic Genome Islands and Microdiversification by Transposons
Source: Front Microbiol. 2022 Feb 21;13:828531. doi: 10.3389/fmicb.2022.828531 (PMC8899612; doi:10.3389/fmicb.2022.828531)
Supplement: Supplementary file 1 [file Data_Sheet_1.doc]

**Supplementary information: Population genomics of *Ferrovum myxofaciens* from mine biostalactites: Genomic 'scrapyards' and microdiversification by transposons**

Kateřina Burkartová^1^, Jiří Dresler^3^, Jakub Rídl^4,5*^, Lukáš Falteisek^2#^

*1 Charles University, Faculty of Science, Department of Philosophy and History of Science*

*2 Charles University, Faculty of Science, Department of Ecology*

*3 Military medical Agency, Military Health institute*

*4 Charles University, Faculty of Science, Department of Zoology*

*5 The Czech Academy of Sciences, Laboratory of Genomics and Bioinformatics, Institute of Molecular Genetics*

** Corresponding author 1*

*telephone: [+42] 737-449-462*

*e-mail: jakub.ridl@img.cas.cz*

*#Corresponding author 2*

*telephone: [+42] 777-643-780*

*e-mail: nealkoholik@seznam.cz*

**Supplementary methods**

**Microbial community composition analysis**

A 253bp fragment of 16S rDNA V4 region was PCR amplified using primers U515F (GTGYCAGCMGCCGCGGTAA) and 806R (GGACTACNVGGGTWTCTAAT) and sequenced on Illumina MiSeq platform in 2x250 bp settings. Sequence data were analyzed by the pipeline SEED v. 2.1.2 (Větrovský et al., 2018). Paired ends were joined by fastq-join (Aronesty, 2011). All of the sequences with mismatches in tags were removed from the dataset. All sequences were clustered into operational taxonomic units (OTUs) and chimeras were deleted using UPARSE implementation in USEARCH 8.1.1861 (Edgar, 2013), with a 97% similarity threshold. Homogeneity of the *F. myxofaciens* OTU was checked by UPARSE at a 99% treshold. The consensus sequence from each OTU was constructed from a MAFFT alignment (Katoh et al., 2013), based on the most abundant nucleotide at each position. The OTUs were identified using Mothur v. 1.42.0 (Schloss et al., 2009) and Silva v132 database (Quast et al., 2013). The identity was confirmed by megaBLAST and BLASTn algorithms against the GenBank nt/nr database for OTUs with abundance over 0.1 % at least in one sample (Table S2).

**References:**

Aronesty E. (2011). ea-utils : "Command-line tools for processing biological sequencing data"; https://github.com/ExpressionAnalysis/ea-utils

Edgar R. C. (2013). UPARSE: highly accurate OTU sequences from microbial amplicon reads. Nature Methods, 10: 996.

Katoh K., Standley D.M. (2013). MAFFT Multiple Sequence Alignment Software Version 7: Improvements in Performance and Usability. Mol. Biol. Evol. 30: 772–780.

Quast C., Pruesse E., Yilmaz P., Gerken J., Schweer T., Yarza P., Peplies J., Glöckner F.O. (2013). The SILVA ribosomal RNA gene database project: improved data processing and web-based tools. Nucl. Acids Res. 41: D590–D596.

Schloss P.D., Westcott S.L., Ryabin T., Hall J.R., Hartmann M., Hollister E.B., Lesniewski R.A., Oakley B.B., Parks D.H., Robinson C.J., Sahl J.W., Stres B., Thallinger G.G., Van Horn D.J., Weber C.F. (2009). Introducing mothur: Open-Source, Platform-Independent, Community-Supported Software for Describing and Comparing Microbial Communities. Appl. Environ. Microbiol. 75: 7537–41.

Větrovský T., Baldrian P., Morais D. (2018). SEED 2: a user-friendly platform for amplicon high-throughput sequencing data analyses. Bioinformatics 34: 2292-2294.


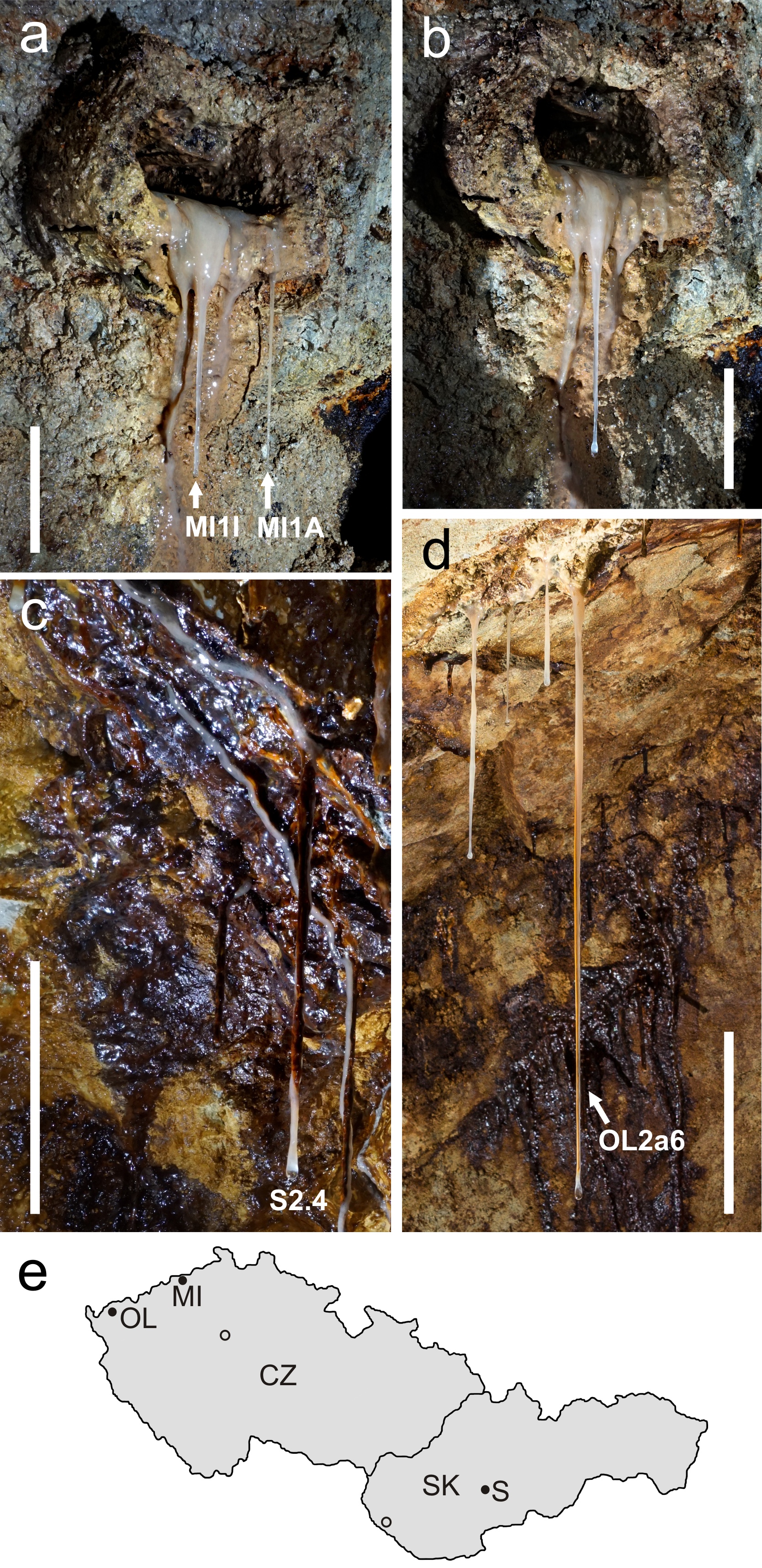


**Figure S1**

**Physical appearance of mine biostalactites sampled for metagenome sequencing.** a. MI1I and MI1A, Lehnschafter mine, Mikulov, Czech Republic; b. the regenerated MI1 biostalactite sampled as MI1III (note no regeneration of MI1A); c. S2.4, Šobov quarry exploratory adit, Banská Štiavnica, Slovakia; d. OL2a6, exploratory adit nr. 112, Oloví, Czech Republic; e. localization of the sample sites. The author of photo d. is Vojtěch Duchoslav.

| **sample** |  | **MI1A** | **MI1I** | **MI1III** | **S2.4** | **OL2a6** |
| --- | --- | --- | --- | --- | --- | --- |
| **sample date** |  | 9.12.2016 | 9.12.2016 | 23.1.2019 | 14.7.2017 | 6.6.2017 |
| **coordinate N** | ° | 50.69104 | 50.69104 | 50.69104 | 48.47111 | 50.25529 |
| **coordinate E** | ° | 13.72136 | 13.72136 | 13.72136 | 18.89955 | 12.54595 |
| **depth b. s.** | m | 60 | 60 | 60 | 30 | 100 |
| **Q** | mL/h | 252 | 137 | 285 | 122 | 9.9 |
| **pH** |  | 2.72 | 2.46 | 2.47 | 2.76 | 3 |
| **EC** | mS/cm | 1.61 | 3.08 | 2.92 | 1.05 | 1.46 |
| **ORP** | mV | NA* | 706 | 686 | 680 | NA |
| **O_2_** | mg/L | NA* | NA | 7.36 | 8.88 | 11.04 |
| **Fe(II)** | mg/L | 40.4 | 15.8 | 49.4 | 8.33 | 22.04 |
| **Fe tot.** | mg/L | 57 | 267 | 226 | 21.1 | 177 |
| **As** | mg/L | 0.77 | 4.85 | NA | <0.005 | 0.59 |
| observed OTUs | | 65 | 13 | 35 | 24 | 43 |
| S-W diversity index | | 0.665 | 0.062 | 0.602 | 0.091 | 0.138 |

* Mixing of water from two sources with contrasting redox state occured in MI1A, the ORP and dissolved O2 values are thus not reliable

**Table S1**

**Basic characterization of biostalactites used for the metagenomic analysis.** Observed OTUs and Shannon-Wiener diversity indices were computed using prokaryotic V4 SSU rDNA amplicon sequences and OTUs determined at a 97% similarity level. All samples were randomly reduced to 10,000 sequences/sample for better comparability (the reduction had negligible effect on the parameters).

| **identification** | **MI1A** | **MI1I** | **MI1III** | **OL2a.6** | **S2.4** |
| --- | --- | --- | --- | --- | --- |
| **Ferrovum myxofaciens** | **87.42** | **99.19** | **88.4** | **98.37** | **98.85** |
| Leptospirillum sp. | 4.94 | 0.08 | 1.83 | 0.01 | 0 |
| Rickettsiales OTU1 | 1.78 | 0 | 2.57 | 0.16 | 0 |
| Metallibacterium sp. | 1.32 | 0.04 | 2.75 | 0.08 | 0.15 |
| Acidithrix ferrooxidans | 0.77 | 0.07 | 1.74 | 0 | 0.01 |
| Leptospirillum cf. ferrodiazotrophicum | 1.23 | 0.03 | 0.3 | 0.11 | 0 |
| Dependentiae OTU1 | 0 | 0 | 0.7 | 0 | 0 |
| Acidisoma sp. | 0.02 | 0 | 0.62 | 0 | 0 |
| **Ferrovum sp.** | **0.13** | **0.39** | **0.01** | **0.03** | **0.01** |
| Elusimicrobia OTU1 | 0.53 | 0.02 | 0 | 0 | 0 |
| Gallionella sp. | 0.06 | 0.08 | 0 | 0.07 | 0.2 |
| Arenimonas sp. | 0.04 | 0.01 | 0 | 0.06 | 0.27 |
| Trichorickettsia cf. mobilis | 0.04 | 0 | 0.3 | 0 | 0 |
| Edaphobacter sp. | 0.06 | 0 | 0.1 | 0.04 | 0.09 |
| Thermogemmatispora sp. | 0.01 | 0 | 0 | 0.15 | 0.08 |
| Holospora sp. | 0.15 | 0.06 | 0.01 | 0.01 | 0.01 |
| Gemmataceae OTU1 | 0.22 | 0 | 0 | 0 | 0.01 |
| Finniella sp. | 0.04 | 0.01 | 0.18 | 0 | 0 |
| Obscuribacterales OTU1 | 0.17 | 0.01 | 0.04 | 0 | 0 |
| Thiomonas sp. | 0 | 0 | 0 | 0.11 | 0.02 |
| others | 1.07 | 0.01 | 0.45 | 0.8 | 0.3 |

**Table S2. Composition of the biostalactite microbial communities.** Percentage of all OTUs seaching at least 0.1 % in one sample are shown. Proposed autotrophs are displayed in blue, members of the genus *Ferrovum* in bold.

| gene | length (AA) | MI1I from bp | MI1I to bp |
| --- | --- | --- | --- |
| LSU ribosomal protein L1p (L10Ae) | 231 | 1912959 | 1913654 |
| LSU ribosomal protein L2p (L8e) | 275 | 1929685 | 1930512 |
| LSU ribosomal protein L3p (L3e) | 214 | 1928111 | 1928755 |
| SSU ribosomal protein S3p (S3e) | 236 | 1931158 | 1931868 |
| SSU ribosomal protein S4p (S9e) | 208 | 1939502 | 1940128 |
| DNA-directed RNA polymerase α subunit | 329 | 1940170 | 1941159 |
| DNA-directed RNA polymerase β subunit | 1356 | 1914934 | 1919004 |
| Translation elongation factor G | 697 | 1924361 | 1926454 |
| Transcription antitermination protein NusG | 178 | 1911914 | 1912450 |
| Protein translocase subunit SecE | 115 | 1911570 | 1911917 |
| Protein translocase subunit SecY | 438 | 1936993 | 1938309 |
| Translation initiation factor 1 | 72 | 1938312 | 1938530 |

**Table S3**

**Characteristics of 12 conserved housekeeping genes used for phylogenetic analysis (Figure 1).**

**Table S4 (table is in a separate excel file)**

**List of genes disrupted by mobile elements.** The numbers in the first column correspond to those in Figure 2a-e. Yellow fields represent disruptions that are present in the reference genome (REF), whereas the blue fields represent gene disruptions found in unassigned reads (ALT). Disruptions verified by PCR (Table 4) and/or manually quantified in Illumina reads (Table 5) are in red. Genes related to motility, cellulose metabolism, and assimilatory nitrate reduction that are missing in some *Ferrovum* sp. strains (Ulrich et al., 2016 and this study) are displayed in bold. Genes with homoplastic disruptions in populations from various sites are underlined.

__________________________________________________________________________________

|  | MI1A | | MI1I | | MI1III | | S2.4 | | OL2a6 | |
| --- | --- | --- | --- | --- | --- | --- | --- | --- | --- | --- |
| gene | nr. | disruption | nr. | disruption | nr. | disruption | nr. | disruption | nr. | disruption |
| aerotaxis sensor receptor | **6** | yes+no | **1** | yes+no | **9** | yes+no | **x** | yes+no* | **x** | no |
| Acetate kinase | **13** | yes+no | **2** | yes+no | **2** | yes+no | **x** | no | **x** | no |
| diguanylate cyclase/phosphodiesterase DGC8 | **21** | no | **5** | yes+no | **25** | no | **x** | no | **x** | no |
| Type II/IV secretion system secretin RcpA/CpaC; disruption 1 | **5** | yes+no | **8** | yes+no | **38** | no | **x** | no | **x** | no |
| Uptake [NiFe] hydrogenase, small subunit HyaA | **x** | no | **x** | yes+no* | **1** | yes+no | **x** | no | **x** | no |
| diguanylate cyclase/phosphodiesterase DGC3 | **x** | no | **14** | no | **12** | no | **1** | yes+no | **x** | no |

* Differences from Nanopore variability search results caused probably by higher sensitivity of the PCR detection

**Table S5**

**The PCR control of disruptions in six selected genes.** The columns marked as “nr.” correspond to numbers in Table S3 and Figure 2. Disruptions not detected in the respective genome by the automated search are marked “x”. Blue fields are for disruptions in unassigned reads whereas yellow for disruptions that are present in the reference genome. The column “disruption” shows result of PCR using primers specific for disrupted and non-disrupted gene sequence, respectively. Genes not present in the given reference genome are displayed in gray.

| **MI1A** | | |
| --- | --- | --- |
| **gene** | **start (bp)** | **# SSV** |
| Putative helicase | 67298 | 68 |
| HNH endonuclease | 151613 | 7 |
| Phage protein (ACLAME 240) | 185045 | 10 |
| Phage protein (ACLAME 240) | 218051 | 14 |
| General secretion pathway protein A | 326795 | 3 |
| Putative helicase | 902915 | 140 |
| diguanylate cyclase/phosphodiesterase | 1215852 | 3 |
| Flagellar motor switch protein FliM | 1581444 | 4 |
| Oligopeptidase A | 1729010 | 4 |
| 5-methyltetrahydrofolate--homocysteine methyltransferase | 1738866 | 6 |
| Ammonium transporter | 1741825 | 3 |
| Circadian clock protein KaiC | 2497357 | 79 |
| **MI1I** | | |
| **gene** | **start (bp)** | **# SSV** |
| Phage protein (ACLAME 240) | 184072 | 7 |
| Phage protein (ACLAME 240) | 217076 | 15 |
| Putative helicase | 905701 | 22 |
| Putative helicase | 906477 | 14 |
| Putative helicase | 906832 | 16 |
| Putative helicase | 908234 | 78 |
| Mobile element protein | 1102663 | 5 |
| Mobile element protein | 1111939 | 4 |
| General secretion pathway protein A | 1181482 | 3 |
| Flagellar motor switch protein FliM | 1587913 | 3 |
| Circadian clock protein KaiC | 2504989 | 79 |
| diguanylate cyclase/phosphodiesterase | 2506526 | 5 |
| **S2.4** | | |
| **gene** | **start (bp)** | **# SSV** |
| Copper/silver efflux RND transporter, membrane fusion protein CusB | 640234 | 40 |
| Copper/silver efflux RND transporter, transmembrane protein CusA | 641457 | 171 |
| Co/Zn/Cd efflux RND transporter, membrane fusion protein, CzcB family | 644643 | 25 |
| Asparagine synthetase [glutamine-hydrolyzing] | 887492 | 4 |
| Mobile element protein | 1011852 | 4 |
| Heavy-chain fibroin (fragment) | 2363499 | 5 |
| Circadian clock protein KaiC | 2364817 | 32 |
| diguanylate cyclase/phosphodiesterase | 2366300 | 12 |
| Mobile element protein | 2399420 | 32 |
| **OL2a6** | | |
| **gene** | **start (bp)** | **# SSV** |
| Putative helicase | 326452 | 4 |
| Mobile element protein | 336492 | 6 |
| Transposase | 342553 | 3 |
| Transposase | 362972 | 6 |
| Mobile element protein | 653337 | 5 |
| Mobile element protein | 885037 | 4 |
| Mobile element protein | 1051974 | 5 |
| Mobile element protein | 1229218 | 3 |
| Mobile element protein | 1229780 | 5 |
| Mobile element protein | 1270417 | 8 |
| Mobile element protein | 1270839 | 4 |
| Mobile element protein | 1290303 | 6 |
| Mobile element protein | 1445998 | 7 |
| small subunit ribosomal RNA - 5 primer truncation | 2359116 | 4 |
| Transposase | 2414843 | 3 |
| Circadian clock protein KaiC | 2626578 | 57 |

**Table S6**

**List of genes, that contain more than two SSVs and were not annotated as hypothetical proteins.** In MI1I genome the same putative helicase which is present in MI1A genome was split into four ORFs by the annotator. Data is not available for MI1III genome.
